# Endoscopic Tattooing Using Indocyanine Green (ICG) Fluorescence for Intraoperative Guidance in Colorectal Surgery: Review of the Literature

**DOI:** 10.3390/cancers18010022

**Published:** 2025-12-20

**Authors:** Fotios Seretis, Antonia Panagaki, Georgios Tziatzios, Paraskevas Gkolfakis, Evdokia Romanou, Vasilis Papastergiou, Andreas Theodorou, Andreas Kapiris, Dimitrios Theodorou, Tania Triantafyllou, Stylianos Kapiris, Konstantina Paraskeva

**Affiliations:** 1 Third Department of Surgery, Evaggelismos General Hospital of Athens, 10676 Athens, Greece; stkapiris@hotmail.com; 2 Department of Gastroenterology, General Hospital of Nea Ionia “Konstantopouleio-Patision”, 14233 Athens, Greece; panagaki.antonia@gmail.com (A.P.); konparaskeva@gmail.com (K.P.); 3 Hepatogastroenterology Unit, Second Department of Internal Medicine-Propaedeutic, Attikon University Hospital, Rimini 1, Chaidari, 12462 Athens, Greece; pgolfakis@gmail.com; 4 Second Department of Surgery, “G. Gennimatas” General Hospital of Athens, 11527 Athens, Greece; evd.rom@gmail.com; 5 Gastroenterology Department, “Evangelismos” General Hospital, 16121 Athens, Greece; vasi.pap@hotmail.com; 6 First Propaedeutic Department of Surgery, National Kapodistrian University of Athens School of Medicine, Hippokrateion General Hospital of Athens, 11527 Athens, Greece; antheo300@gmail.com (A.T.); dimitheod@netscape.net (D.T.); t_triantafilou@yahoo.com (T.T.); 7 School of Medicine, University of Patras School of Health Sciences, 26504 Rio Patras, Greece; kapirisandrew@gmail.com

**Keywords:** indocyanine green, ICG, tattoo, colorectal cancer, surgery, endoscopy

## Abstract

Background and Objectives: Accurate endoscopic lesion localization is crucial for planning and performing curative-intent surgery in colorectal cancer management. The use of indocyanine green (ICG) has been described as a novel alternative for colorectal lesion marking. Materials and Methods: We have performed a systematic review of the literature on the use of ICG-based tattooing for patients with colorectal cancer undergoing surgery. Results: A total of 19 studies were identified. Seven studies reposted the rates of successful intraoperative localization following ICG tattooing. Additionally, six studies provided detailed descriptions of the administration protocols, including both timing and dosage. A total of twelve studies described the utility of ICG-based tattooing for subsequent lymphadenectomy and its oncologic implications. Lymphadenectomy under fluorescent guidance was associated with increased lymph node yields as well as a change to the surgical lymphadenectomy plan in a significant proportion of patients. Conclusions: ICG-based endoscopic tattooing has demonstrated significant value in facilitating precise lesion localization during curative-intent colorectal surgery. Moreover, its use has been extended to guiding lymphadenectomy, with reports indicating improved lymph node yields. Nevertheless, further research is required to standardize protocols and address existing limitations.

## Simple Summary

Indocyanine green (ICG) has been recently described in endoscopic tattooing of colorectal lesions treated with curative-intent surgery combined with near-infrared laparoscopic and robotic platforms. Our systematic review of the literature has focused on its utility in intraoperative tumor identification rates, and in identifying tumor-associated lymphatic drainage and performing radical lymphadenectomy. We have also described protocols used in timing and dosages used in ICG-based tattooing as well as associated adverse events. Significant potential benefits in all aforementioned aspects have been noted. However, existing data suffer from significant limitations.

## 1. Introduction

Endoscopic lesion localization has been consistently suggested as key in surgical management of colorectal lesions, whether this includes significant polyps with suspicion of submucosal invasion, technically not endoscopically removable lesions, or overt colorectal cancers. The most recent version of the European Society of Gastrointestinal Endoscopy (ESGE) guidelines for colorectal polypectomy recommends tattooing of all lesions that are referred for subsequent surgery, suggesting that the marking should be placed 3–5 cm distal to the lesion [1]. Despite reports that simple optical evaluation can reliably identify sites of scarring on a post-polypectomy surveillance colonoscopy after endoscopic mucosal resection (EMR), thus obviating the need for tattooing [2], this might not be the case for lesions being referred for surgery. This might especially be the case if one considers the emerging concepts of the Significant Polyps and Early Colorectal Cancer (SPECC) program from the United Kingdom [3], which are also beginning to spread across other parts of the globe, and call a priori for a multidisciplinary assessment of significant polyps or early cancers from a team comprising surgeons, interventional gastroenterologists, and radiologists at the very least [4]. Results from an international Delphi Consensus on indications for endoscopic tattooing in the colon [5] suggest that tattooing should always be considered in the case of lesions undergoing endoscopic resection with concurrent suspicion of deep submucosal invasion, and also in the case of lesions not undergoing endoscopic resection in which the lesion is deemed difficult to detect in subsequent endoscopy with curative intent. Notably, there was also clear consensus that the tattoo should never be directly endoscopically injected into or underneath the lesion.

Another factor to consider is that endoscopic tattooing has its limitations and is often found to err in accurately localizing lesions undergoing surgical resection. A recent post-hoc analysis from a prospective study [6] reported an overall 79.2% accuracy for endoscopic localization pre-operatively when compared to the gold standard of surgical (intraoperative) localization, especially for lesions in the rectum. For lesions in the colon, patient age above 65 years and insertion time of more than 10 min were also associated with significant localization error rates. This might have significant implications, especially when considering the vastly different surgical approaches for lesions in the rectosigmoid/upper rectum area and lesions in the mid/distal third rectum area. Moreover, the advent of minimally invasive surgery and robotics in colorectal cancer surgery only complicate matters, especially if one considers the lack of tactile feedback with currently used robotic platforms or the reduced tactile feedback with laparoscopic instruments. Inaccuracy in colonoscopic localization has been reported at a rate of up to 19% in another study [7], which translated to a 6.3% on-table change in surgical planning. Also corroborating are the results from a recent systematic review and meta-analysis on preoperative localization of colorectal lesions, which reported the superiority of tattooing vs. non-tattooing in reducing localization errors (9.5% vs. 15.4%) [8]. This, unfortunately, still leaves a significant number of patients (almost 10% in this report) that have their lesions erroneously located.

Accurate lesion localization becomes of paramount importance especially in rectal cancer patients, as an ever-increasing proportion of them undergo neoadjuvant chemo radiotherapy. The concepts of total neoadjuvant treatment and organ preservation strategies in good responders [9,10] call for accurate lesion localization for active surveillance and, more importantly, for distal margin clearance in those patients that end up in surgery, either as salvage after recurrence or due to poor response to initial treatment [11].

Recently, indocyanine green (ICG) (Verdye solution 5mg/mL, Diagnostic Green, Diagnostic Green GmbH, Gernany) has gained increasing use in colorectal surgery as a means of utilizing its fluorescent properties combined with infrared-light emitting laparoscopes to facilitate image-guided operations [12]. ICG-based fluorescent angiography is increasingly being used in colorectal surgery as a means of intraoperative colonic perfusion assessment prior to anastomosis creation with the heralded promise of anastomotic leak reduction [13]. Although multiple studies have suggested a trend towards lower anastomotic leak rates, this has not been clearly demonstrated [14,15], and at present it is still unclear which patient groups benefit the most. Most interestingly, ICG has been used to tattoo lesions for subsequent intraoperative intra-operative localization. We conducted a comprehensive review of the literature to explore the potential applications of ICG-marking of lesions in colorectal surgery, including timing of application, technical considerations, efficacy, and associated limitations. The growing adoption of minimally invasive surgical techniques, together with the limitations of traditional endoscopic tattooing methods, highlights the increasing relevance of ICG fluorescence technology for intraoperative lesion localization.

## 2. Materials and Methods

We have performed a comprehensive review of the literature in “Pubmed” and “Embase” databases using search terms: “ICG AND tattoo AND colorectal”, “ICG and tattooing AND endoscopy”, “ICG AND Endoscopic AND tattoo”, “ICG and lymphadenectomy AND colorectal cancer”. References from retrieved references were also manually reviewed for additional sources of data. Two independent reviewers (FS and AP) reviewed the literature independently. In cases of dispute a senior author (KP) made the final decision on inclusion/exclusion of the relevant study. We refrained from performing a systematic review according to strict PRISMA (Preferred Reporting Items for Systematic reviews and Meta-Analyses) guidelines, as the focus of our work was to provide readers with a unified perspective on the use of ICG in the endoscopic tattooing of lesions, with emphasis on its clinical implications, potential to affect oncologic outcomes, as well as its limitations, and to try to unify the relevant concepts. We therefore thought that this purpose could be better served by a narrative review format, as studies in the field are few, do not follow a unified methodology, and thus could not be grouped together. Moreover, the way we have chosen to subgroup our results could also not be served by the format of a systematic review.

### 2.1. Inclusion Criteria

- Studies in English
- Studies on patients with lesions in the colonic and/or rectal area undergoing surgery with curative intent
- Studies in which colorectal lesions were marked endoscopically and were identified in subsequent surgery using infrared-emitting cameras
- Studies reporting on accuracy of lesion localization using ICG
- Studies on oncologic implications of ICG-based lesion marking including lymph node dissection
- Studies on technical notes/protocols used for ICG-based endoscopic tattooing

### 2.2. Exclusion Criteria

- Studies in languages other than English
- Single case reports or studies with up to 10 patients included
- Studies with unclear methodology
- Studies with endpoints other than the ones described in the inclusion criteria
- Studies with ICG injection in places other than submucosa, for example subserosal or intravenous ICG administration
- Studies with ICG injection focusing on identification of sentinel lymph nodes

### 2.3. Studies Were Grouped Together According to the Following Sections

- Studies on accuracy of ICG-based localization compared to the gold-standard of surgical localization
- Studies on protocols and technical details of ICG-based endoscopic lesion marking
- Studies on ICG-guided lymph node dissection after endoscopic tattooing

## 3. Results

**Figure 1:**
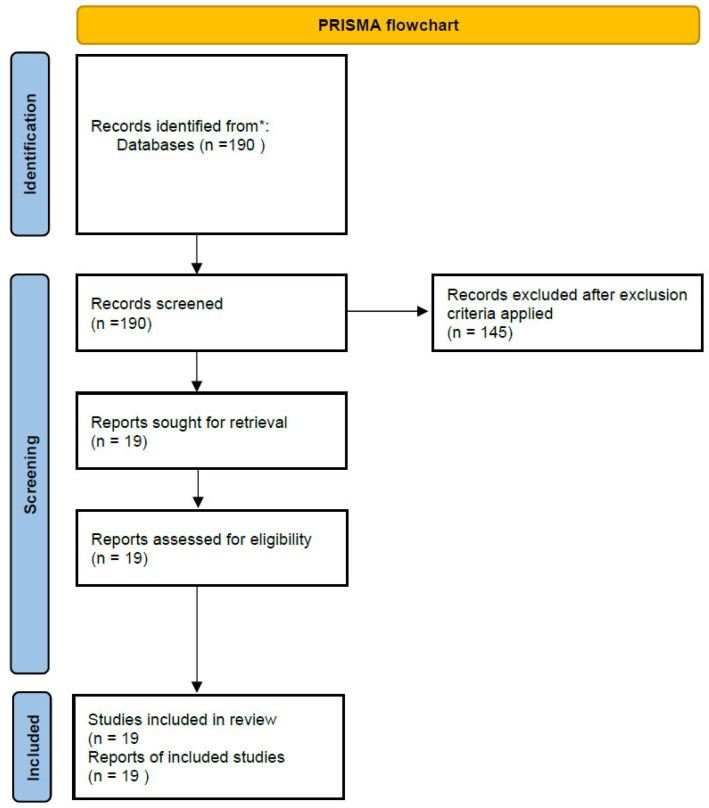
PRISMA flowchart of the literature. (* corresponds to Pubmed and Embase databases).

An initial search yielded 190 articles. After checking abstracts for relevance, 145 articles were excluded. After duplicate exclusion and application of exclusion criteria, a number of articles were also excluded. After full-text review, 16 studies were selected to be included in the analysis. A further three eligible articles were included after manual checking of references, yielding a total of 19 articles included. The results of the literature search are graphically depicted in a flowchart (Figure 1). All included references were grouped according to three subheadings, namely ICG tattooing of lesions, ICG-guided lymphadenectomy, and ICG tattooing dosage and protocols.

### 3.1. ICG-Tattooing of Lesions

Seven studies [16,17,18,19,20,21,22] were identified as including patients undergoing laparoscopic resection for colorectal cancer, with only one out of the eight also including patients undergoing resection in an open fashion. With regards to the rates of intraoperative tumor visualization, excellent rates were reported in most studies, with rates of 93–100% intraoperative tumor marking. Visualization rates intraoperatively dropped significantly in cases where ICG was injected at an interval of more than 7 days preoperatively. In the report from Satoyoshi et al. [16], ICG-based tattoo of a lesion 7–9 days preoperatively yielded a subsequent intraoperative localization rate of 60%, which dropped to 0% for lesions marked more than 10 days before the index operation. Similarly, results from Miyoshi et al. [17] reported rates of 20% intraoperative lesion localization for ICG tattoos placed more than 9 days preoperatively.

With regards to adverse reactions from ICG-tattooing, only one patient developed abdominal pain related to the procedure in the study from Kim et. al. [18], and one patient had ICG spillage in the peritoneal cavity found during the subsequent operation in the study from Miyoshi et al. [17]. No allergic reactions were reported in any of the eight studies. All results regarding intraoperative tumor localization rates and adverse reactions are summarized in Table 1.

### 3.2. ICG Tattooing Dosage and Protocols

Details of the timing of ICG-based lesion tattooing were available in six out of the seven studies. Only in the study from Nagata et al. [20] and Kim et al. [18], was the ICG tattoo placed within 4 days preoperatively from the index surgery. The study from Miyoshi et al. [17] reported a wide time interval for ICG tattooing, ranging from 1–73 days preoperatively. Studies from Watanabe et al. [19], Satoyoshi et al. [16], and Lee et al. [22] included patients with ICG injection more than 7 days preoperatively.

With regards to the specifics of tattooing procedures per se, a wide variation among techniques was noted in most studies. In most studies, ICG was injected submucosally at cardinal locations around the tumor at different volumes and concentrations, with or without normal saline. An exception is the study from Kitagawa et al. [21], which reported the use of a fluorescent ICG-coated clip for lesion marking, rather than ICG submucosal injection. Essentially, this study describes an approach for ICG-based lesion marking without tattooing, but rather the combination of endoscopic clipping with ICG-based technology, thus being an endoscopic ICG-based localization method without the use of tattooing. The results from studies regarding timing and protocols for ICG-based tumor localization combined with endoscopy are presented in Table 2.

### 3.3. ICG-Guided Lymphadenectomy

A total of twelve studies [23,24,25,26,27,28,29,30,31,32,33,34,35] report on the effect of ICG-based endoscopic tattooing on subsequent lymphadenectomy during surgery. Relevant articles were categorized according to the identification of tumoral lymphatic drainage intraoperatively, the effect on lymph node yields during surgery, the intraoperative change in the lymphadenectomy plan based on ICG-lymphatic mapping, and finally oncologic outcomes, including local recurrence rates and/or survival data.

#### 3.3.1. Use of ICG-Tattoo for Tumoral Lymphatic Drainage Intraoperative Identification

Regarding the use of ICG tattooing for intraoperative lymphatic drainage identification, three studies were identified [23,24,25]. Firstly, in a study by Cahill et al. [23], preoperative endoscopic submucosal ICG injection was utilized for intraoperative assessment of tumor lymphatic drainage in a small cohort of 14 patients, with authors concluding that ICG tattooing could be utilized for confirmation of lymphatic drainage patterns in the mesocolon.

Moreover, the study from Ahn et al. [24] reported on the use of different doses of ICG submucosally injected 12–18 h before surgery, ranging from a standard dose of 2.5 mg/mL down to the minimum dose of 0.2 mg/mL, with the outcome being successful tumor localization and lymph node mapping. The authors reported higher success rates for doses between 0.5–1 mg/mL and the use of multiple ICG submucosal injections in preoperative colonoscopy, as well as for patients with lower Body Mass Index (BMI), yielding successful fluorescent-based lymph node mapping in 84% of patients using this ICG dose. Interestingly, despite an increased number of lymph nodes harvested in the group with successful ICG-based localization, the number of involved lymph nodes did not differ among the groups of successful and unsuccessful ICG-based localization. In the same study, surgeons performed radical lymphadenectomy at the D3 level for all patients, removed any fluorescent positive lymph nodes outside the D3 lymphadenectomy plane, and used the lack of ICG fluorescence in the surgical resection bed as documentation of completeness of cancer resection. In the subgroup of patients undergoing D3-level lymphadenectomy, although a higher lymph node yield was achieved with the use of ICG, the rate of involved D3-level lymph nodes did not exert any statistical difference between the ICG use and non-ICG use groups. On the contrary, in another report [25], ICG endoscopic tattoo-based intraoperative lymph visualization was achieved in only 75% of patients, with lower visualization rates, namely 71.5%, being reported for patients with more advanced disease stages.

#### 3.3.2. ICG-Based Tattoo on Lymph Node Yields During Surgery

Four studies reported on lymph node yields in patients undergoing laparoscopic resection for colorectal cancer with endoscopic ICG lesion tattooing.

Park et al. [26] reported a higher mean lymph node yield with ICG use for patients undergoing laparoscopic right hemicolectomy with radical D3 dissection, with no difference, however, in the number of involved nodes between the ICG use vs. non-use groups. Results from a randomized controlled trial, including patients undergoing laparoscopic surgery and radical lymph node dissection at the D3 level [27], reported an increased lymph node yield for patients with ICG tattoo-based lymphadenectomy vs. the standard technique, with no difference, however, in the rates of positive lymph nodes at the D1, D2, or D3 level. In patients with mid-low rectal cancers according to the results of a prospective non-randomized controlled study, the use of ICG submucosal tattooing to guide intraoperative lymphadenectomy also led to an increased lymph node yield [28].

Finally, the study by Goo et al. [29] reported increased lymph node yield due to the use of ICG tattooing only in patients with early-stage colorectal cancer, while no advantage over the standard technique was demonstrated for more advanced stages of the disease. Similar results with increased lymph node yields for rectal cancer patients are reported by Zhoo et. al. [30].

#### 3.3.3. ICG-Based Change of Lymphadenectomy Plan

Three studies reported on the rate of intraoperative change in the surgical dissection plan for lymphadenectomy during curative-intent surgery, based on lymphatic drainage characterization after tumor ICG tattooing with ICG submucosal injection.

In an interim analysis from the GREENLIGHT study [31] on 70 out of the total 100 patients undergoing robotic colonic or rectal resection and receiving preoperative injection of ICG submucosally 1 or 3 days preoperatively, intraoperatively the lymphadenectomy was changed in 50% of patients, mostly being an excision of lymphatic station outside the originally planned lymph node basin in 41.4% of patients. In the same study the authors reported a trend, although not statistically significant, for increased aberrant lymph node detection in patients receiving ICG tattooing within 24 h from surgery vs. those receiving it 3 days preoperatively. Similarly, Nishigori et al. [32] reported a change in the surgical plan for lymphadenectomy in 23.5% of patients.

In another report [33] concerning submucosal injection of ICG in 20 patients with flexural tumors (10 hepatic flexure and 10 splenic flexure tumors) undergoing laparoscopic complete mesocolic excision with central vascular ligation, the authors reported a 95% rate for intraoperative lymphatic flow visualization as well as an intraoperative ICG-guided lymphadenectomy area in 5/20 (25%) patients.

#### 3.3.4. ICG-Based Endoscopic Tattooing Affecting Subsequent Oncologic Outcomes

Only two studies reported on oncologic outcomes, such as recurrence rates or survival outcomes.

With regards to oncologic outcomes for ICG-guided lymphadenectomy for rectal cancer, Watanabe et al. [34] reported on 172 patients with middle-lower third rectal cancers undergoing laparoscopic lateral lymph node dissection. In this report, patients receiving submucosal ICG-injection and subsequent ICG-guided lymphadenectomy had lower local recurrence rates than their counterparts not receiving fluorescent-imaging guided lymphadenectomy, with no difference, however, in 3 year disease-free and overall survival. On the contrary, a recent report from Chen et al. [35] on rectal cancer patients reported an increased lymph node yield for ICG tattoo-guided surgery vs. the standard technique. Also, significantly in favor of the ICG group was the increased rate of detection of involved inferior mesenteric artery lymph node stations (apical nodes), thus providing improved pathologic staging, according to the authors.

## 4. Discussion

Endoscopic lesion marking based on ICG technology has recently emerged as an alternative to classic Indiavink stain-based tattooing performed by endoscopists. The heralded promise is that such a tattoo might be visible through infrared-emitting laparoscopic or robotic platforms, thus providing real-time intraoperative navigation. In our systematic review of the literature, we have found rates of intraoperative tumor localization at ranges above 92% up to 100% among the various studies, if ICG is submucosally injected through preoperative colonoscopy, provided that the ICG tattoo was performed within 4 days from index operation. The rate of successful intraoperative tumor localization was significantly reduced when the tattooing procedure was performed more than 4 days prior to the index operation. From a technical perspective, it is also important to note the considerable heterogeneity reported across studies regarding both the volumes of ICG administered submucosally and the precise injection technique, including whether the agent was delivered peritumorally or directly into the tumor itself.

ICG tattooing appears to be safe with virtually no adverse events reported in the literature. This contrasts with the well-described complications associated with India-ink, including cases of intraperitoneal leakage leading to inflammatory reactions, which subsequently complicate surgical dissection and impede accurate tumor identification [36].

Conversely, because the visibility of ICG markings diminishes significantly after approximately 10 days, these cases may still necessitate the use of conventional India ink tattooing.

Aside from tumor localization, the main strength and expanded utility of ICG-based lesion tattooing lies in its ability to serve as an intraoperative roadmap for lymph node dissection. In essence, lesion marking also marks the lymphatic basin draining the tumor area, thus providing an individualized map of tumor draining areas. The studies included in this review report a change in operative strategy regarding lymphadenectomy in more than 23% of cases with reported rates reaching up to 50%. The interim analysis of the GREENLIGHT study included patients undergoing right-sided, left-sided, and anterior resections, thus the change in the lymphadenectomy plan might apply to a significant proportion of patients with colorectal cancer. From an anatomic perspective, and in light of emerging data on anatomic variations in both the right and left hemicolon [37,38,39] that might also involve aberrant lymphatic drainage pathways, these findings become increasingly important. In the case of flexural tumors, the considerations become critical. Notably, nearly 50% of patients underwent ICG-based modification of the lymphadenectomy strategy in a single study [33] that exclusively included flexural tumors. Further investigations with large cohorts as well as analogous evaluations in transverse colon tumors are warranted and may yield findings of substantial clinical significance. Importantly the report by Watanabe et al. (33) conceptually integrates the principles of complete mesocolic excision with ICG-tattooing guidance, demonstrating that ICG can delineate a tumor-specific mesocolic envelope along embryologically determined potentially dissectible surgical planes. In our view, advancing the quality of colorectal cancer resection may depend on unifying complete mesocolic excision with intraoperative ICG-guided tumor marking to reliably achieve surgical resection radicality, which translates to better oncologic outcomes for patients.

Another important point regarding ICG-based lymphadenectomy is the increased lymph node yields achieved through fluorescent guidance. In the present study we only focused on results after submucosal ICG injection via endoscopy. However, a significant body of literature reports on sub-serosal ICG injection. This, in our opinion, might lead to inaccuracies especially in earlier stage cancers, which appear to benefit the most from ICG-tattooing with regards to lymph node yields. Another point to be taken into consideration is that although increased lymph node yields were reported in many studies, only in one study from Chen et al. [35] were increased rates of involved lymph node detection reported. In our opinion, focusing solely on the number of lymph nodes retrieved is a point of clinical relevance, but should not be regarded as the sole quality indicator of an adequate cancer resection, as we do know that other factors such as the ratio of involved lymph nodes over total lymph nodes resected, tumor deposits positivity status, lymphovascular or perineural invasion, as well as molecular features of the tumor may be even more important prognostically [40,41]. We did not identify any study that included all of these prognostically important factors and incorporated them into an ICG-based lymphadenectomy therapeutic scheme. Moreover, other factors, such microsatellite stability status or delivery of neoadjuvant treatments, have not been studied in the context of ICG-based lymphadenectomy. We do believe that this should be a focus of further studies.

A significant limitation in generalizing of our results is the fact that most studies come from Asian centers, thus they may not be so easily applicable to Western populations. Notably, when reviewing data on ICG-guided lymphadenectomy in colorectal cancer, increased BMI was found to negatively affect the ability to detect fluorescent lymphatic channels and stations in the mesocolon.

From a technical point of view, although feasible, submucosal injection of ICG by endoscopy in most studies is performed in the preoperative setting (usually 1–5 days before the index procedure). When the injection was performed more than 1 week preoperatively, then the subsequent ability to localize the tumor intraoperatively was reported to be poor. This might create challenges from a resource and administration point of view, because one more endoscopic procedure is required for each patient. This is in sharp contrast to the use of India-ink stain, which can be applied even weeks before the index procedure. Also, in the technical aspects of ICG-based lesion tattooing, it should be noted that a significant heterogeneity of protocols and dosages were noted in the literature, thus making direct comparisons between studies slightly problematic. The unique report from Kitagawa et al. on the use of fluorescent endoscopic clips might be a solution to overcome some of the aforementioned difficulties, but since this is the only report in the literature, more clinical data and validation from future studies, as well as more information regarding cost-effectiveness, should be available before making any recommendations. However, conceptually, this seems to be an interesting approach to bringing together the best of two worlds, namely ICG-based intraoperative navigation and lesion marking without need for repeat endoscopic session in the immediate preoperative period.

There are, though, indications for endoscopic lesion marking where abandoning the practice of India ink-based tattooing might not be the optimal strategy. From the surgical oncology point of view, and the ever-increasing neoadjuvant treatments in the treatment primarily of rectal cancer, this might have important implications. Using ICG tattooing to mark a lesion for a patient with rectal cancer undergoing months of neoadjuvant chemo-radiotherapy or even total neoadjuvant therapy might therefore not be prudent [42,43]. Furthermore, we did not encounter any data on ICG-based tattoos for patients undergoing organ-preservation strategies who might, at some point of their treatment, either have a complete clinical response, or on the contrary might undergo either local excision or radical surgery with curative intent, thus requiring lesion localization for endoscopic follow up or resection, respectively. Moreover, no data on ICG-based tattooing were encountered for patients undergoing advanced polypectomy such as endoscopic mucosal resection (EMR) or endoscopic submucosal dissection (ESD), which might require either surveillance of the polypectomy site or further endoscopic procedures based on initial histopathologic response or recurrence.

Although the study from Cahill et. al. [23] focused on ICG-based tattooing and subsequent sentinel lymph node identification, other studies have failed to demonstrate the feasibility of the sentinel lymph node dissection [44]. We deliberately opted to not include studies on sentinel lymph node identification using ICG-tattooing, as we felt that the concept of sentinel lymph nodes has not yet gained widespread acceptance in the surgical community at the time of this publication, which is also supported by a recent systematic review [44]. However, we did come across studies on sentinel lymph identification using ICG and laparoscopic or robotic platforms in colorectal cancer surgery [45]. Therefore, this is an area of active research.

Unfortunately, there are some further limitations in our work. Firstly, most studies are retrospective in nature, thus their conclusions might have an inherent risk of bias. The retrospective nature of the included studies, the wide variability in the exact protocols for ICG tattooing and timing of marking in relation to index surgery, as well as the lack of common primary endpoints in included studies precluded us from performing a meta-analysis of our results.

Another limitation of our work is the wide variation in the protocols employed across the various studies in ICG-based tattooing. Differences in dosages of ICG used and in the timing of administration in relation to operation preclude any direct comparison between results, especially regarding rates of intraoperative tumor identification and lymphatic drainage mapping. Therefore, the generalizability of the conclusions might be reduced.

Also limiting is the fact that only a few studies focus on the quality of surgical specimens, namely pathologic grading of the surgical specimen quality, especially when considering the concept of complete mesocolic excision for colonic cancers [46]. The data on ICG-based tattooing of lesions without accounting for the quality of surgical resection, namely the quality of mesocolic excision, central ligation of feeding vessels, and adequate lymph node harvest, do not provide the full picture on potential benefits and limitations. Focusing only on the number of lymph node yields as an important endpoint suffers from limitations in capturing to the full extent what constitutes a proper oncologic resection. With regards to other clinically meaningful endpoints, namely a change in the lymphadenectomy plan and intraoperative lymphatic drainage identification, only three studies were identified for each of these endpoints, respectively. Moreover, the included studies lack long-term follow up, thus the oncologic implications of the technique might not be fully understood at present. Ideally, data from randomized controlled trials, including control of surgical quality metrics as well as long-term follow up, might help establish the use of ICG in tattooing of colorectal lesions being surgically treated.

However, the strength of our results lies in the attempt we made to unify the concepts of ICG-based endoscopic tattooing with intraoperative lesion identification, the potential benefits for lymphadenectomy, and a detailed description of existing protocols and dosages. Using ICG tattooing in marking the tumor directly and its associated lymph node basin to be removed en bloc alongside embryologically defined planes might, in the future, unify the concepts of complete mesocolic excision and tumor-specific lymphadenectomy. However, initial positive results should not inflate the perceived benefits of the method, especially from the oncologic point of view, before more solid data become available in the future, especially from randomized controlled trials.

## 5. Conclusions

ICG lesion tattooing with endoscopy is a promising modality with excellent rates of intraoperative tumor identification at subsequent surgery with minimal adverse effects. A significant variation exists among studies regarding the timing and dosage of ICG administration. However, ICG tumor labeling may assist in tumor lymphatic drainage characterization, thus leading to increased lymph node yields and a change in the intraoperative lymphadenectomy plan in a significant proportion of patients. It is not currently established how this translates to better quality surgery and better cancer related outcomes, as studies are few and other prognostically important factors are not incorporated in current studies. The existing literature is mainly retrospective in nature, thus being prone to potential biases. Further studies in the field are required to explore all the possibilities for successful and widespread adoption of this technique into modern colorectal cancer treatments.

## Figures and Tables

**Table 1:** Localization rates and adverse reactions with ICG-based endoscopic tattoo.

Study	N. of Patients	Operation	Outcomes Intraoperative Tumor Localization	Adverse Reactions Related to ICG
Watanabe et al. 2017 [19]	80 patients	Laparoscopic colorectal resection	93.8%	None
Satoyoshi et al. 2021 [16]	165 patients	Laparoscopic colorectal resection	100% within 6 days preoperatively 60% for 7–9 days preoperatively 0% for ≥10 days	None
Nagata et al. 2016 [20]	24 patients	Laparoscopic colorectal resection	100% Interesting 10/24 ICG positive but negative for India Ink	None
Kitagawa 2023 [21]	305 (86 fluorescent clip marking vs. 219 tattooing with India Ink)	Laparoscopic colorectal resection	93.02% for fluorescent clip marking vs. 75.80% for India Ink marking	None
Kim et al. 2020 [18]	227 (149 in ICG group vs. 78 patients India Ink)	Laparoscopic (60.4%) or open (39.6%) colorectal resection	96% for ICG vs. 98.7% for India Ink	One patient in ICG abdominal pain related to tattooing
Miyoshi et al. 2009 [17]	40 patients	Laparoscopic or open colorectal resection	-100% for patients with injection within 1–8 preoperatively -20% for patients receiving tattoo within more than 9 days	One patient with ICG spillage in peritoneal cavity
Lee et al. 2018 [22]	174 patients	Laparoscopic colorectal surgery	-95% visualization within 2 days preoperatively vs. 40% if between 3–14 days preoperatively	None

**Table 2:** Timing of ICG tattooing and protocols for injection used.

Study	N. of Patients	Timing of ICG Injection	Protocol for ICG-Based Tumor Localization
Watanabe et al. 2017 [19]	80 patients	Better visibility for injection <7 days preoperatively vs. ≥10 days	0.5 mL of ICG submucosal injection (2.5 mg/mL) location related to tumor?
Satoyoshi et al. 2021 [16]	165 patients	-Within 6 days preoperatively -Within 7–9 days preoperatively -More than 10 days preoperatively	1 mL total of ICG submucosal injection opposite to tumor. Total dose 0.5 mg of ICG (5 mg/mL)
Nagata et al. 2016 [20]	24 patients	-Within 4 days preoperatively	Indocyanine green + normal saline submucosal injection on the other side of lesion preoperatively
Kitagawa et al. 2023 [21]	305 (86 fluorescent clip marking vs. 219 tattooing with India Ink)	Not specified	Fluorescent clip marking (Zeoclip FS^®^) placed preoperatively
Kim et al. 2020 [18]	227 (149 in ICG group vs. 78 patients India Ink)	1–4 days preoperatively for ICG cases	ICG submucosal injection at three sites (0.5 mL of ICG injection) circumferentially around tumor
Miyoshi et al. 2009 [17]	40 patients	1–73 days (median 4 days)	1 mL of ICG (12 mg/mL) + 4 mL of normal saline submucosal injection adjacent to the lesion
Lee et al. 2018 [22]	174 patients	0–14 days preoperatively	1–1.5 mL of ICG + saline submucosal injection at two to three sites opposite the lesion

## Data Availability

All data are available from authors upon reasonable request.

## Abbreviations

The following abbreviations are used in this manuscript:

BMI	Body Mass Index
ICG	Indocyanine green
EMR	Endoscopic mucosal resection
ESD	Endoscopic submucosal dissection
ESGE	European Society of Gastrointestinal Endoscopy
AJCC	American Joint Commission on Cancer
MSI	Microsatellite Instability
